# Comparison of the specificity of antibodies to VAR2CSA in Cameroonian multigravidae with and without placental malaria: a retrospective case–control study

**DOI:** 10.1186/s12936-015-1023-6

**Published:** 2015-12-01

**Authors:** Anna Babakhanyan, Rui Fang, Andrew Wey, Ali Salanti, Grace Sama, Canisia Efundem, Robert J. I. Leke, John J. Chen, Rose G. F. Leke, Diane W. Taylor

**Affiliations:** Department of Tropical Medicine, Medical Microbiology and Pharmacology, John A Burns School of Medicine, University of Hawaii at Manoa, 651 Ilalo Street, BSB 320, Honolulu, HI 96813 USA; Faculty of Medicine and Biomedical Research, Biotechnology Centre, University of Yaounde 1, Yaounde, Cameroon; Department of Immunology and Microbiology, Centre for Medical Parasitology, University of Copenhagen, Copenhagen, Denmark

**Keywords:** Antibodies, Avidity, VAR2CSA, Placental malaria

## Abstract

**Background:**

Antibodies (Ab) to VAR2CSA prevent *Plasmodium falciparum*-infected erythrocytes from sequestrating in the placenta, i.e., prevent placental malaria (PM). The specificity of Ab to VAR2CSA associated with absence of PM is unknown. Accordingly, differences in the specificity of Ab to VAR2CSA were compared between multigravidae with and without PM who had Ab to VAR2CSA.

**Methods:**

In a retrospective case–control study, plasma collected from Cameroonian multigravidae with (n = 96) and without (n = 324) PM were screened in 21 assays that measured antibody levels to full length VAR2CSA (FV2), individual VAR2CSA DBL domains, VAR2CSA domains from different genetic backgrounds (variants), as well as proportion of high avidity Ab to FV2.

**Results:**

Multigravidae with and without PM had similar levels of Ab to FV2, the six VAR2CSA DBL domains and different variants, while the proportion of high avidity Ab to FV2 was significantly higher in women without PM at delivery (p = 0.0030) compared to women with PM. In a logistic regression model adjusted for gravidity and age, the percentage of high avidity Ab to FV2 was associated with reduced likelihood of PM in multigravidae. A 5 % increase in proportion of high avidity Ab to FV2 was associated with a nearly 15 % lower likelihood of PM.

**Conclusion:**

Ab avidity to FV2 may be an important indicator of immunity to PM.

**Electronic supplementary material:**

The online version of this article (doi:10.1186/s12936-015-1023-6) contains supplementary material, which is available to authorized users.

## Background

In pregnant women, *Plasmodium falciparum*-infected erythrocytes (IE) express the VAR2CSA malarial adhesin that mediates binding of IE to placental chondroitin sulfate A (CSA) [1, 2]. Pathology results from the accumulation of IE causing a condition called placental malaria (PM). PM endangers the health of pregnant women and the developing fetus [1]. Antibodies (Ab) to VAR2CSA play an important role in protection from the adverse outcomes of PM in women living in malaria-endemic areas. Over successive pregnancies, women produce Ab to VAR2CSA that inhibit the binding of IE to CSA in vitro [3, 4], reduce maternal anaemia [5], lower placental parasitaemia at delivery [6, 7], increase the length of gestation [8], and improve infant birth weight [8]. VAR2CSA is a primary vaccine candidate for PM and is currently under clinical investigation [9]. Since full-length VAR2CSA is 350 kDa, researchers are exploring various sequences of VAR2CSA Duffy-binding-like (DBL) domains to develop a vaccine, including the N-terminal ID1-ID2a sequence that contains the CSA-minimal binding site [10], DBL1-2 and DBL3x [11] and DBL1x-DBL3x [12] and C-terminal domains 4 (DBL4ε) and 5 (DBL5ε) [13]. In addition to the selection of VAR2CSA DBL domain(s) for inclusion in a vaccine, other Ab properties may affect vaccine efficacy, such as the ability to inhibit IE binding to CSA [4, 6, 10–12], ability to mediate opsonic phagocytosis [5, 14–17], a broad Ab repertoire to multiple VAR2CSA DBL domains [18] and high avidity Ab to the full-length VAR2CSA (FV2) early in pregnancy [19]. A vaccination strategy that could elicit long-term immunity with cross protection against different *P. falciparum* strains will be of great value and have an impact on global public health. Long-term humoral immunity is produced in germinal centers [20], where antigen-stimulated B cells undergo clonal expansion, and immunoglobulin class-switching, somatic hypermutation and clonal selection, leading to affinity maturation of Ab [21, 22]. As the B cell response matures, Ab levels increase, more epitopes are recognized and Ab quantity and quality increase. The pattern of Ab maturation and its role in Ab effector responses against PM in low-transmission areas remain unexplored.

This study examined specificity, including quantity and quality of the immune response to VAR2CSA, among Cameroonian multigravid women living in malaria low-transmission areas. Archival samples used were collected before implementation of intermittent preventive treatment (IPT) with sulfadoxine-pyrimethamine and insecticide-treated bed nets; thus, women developed significant levels of immunity to PM. However, a proportion of multigravidae, even after three pregnancies, had PM at delivery. Therefore, it is plausible that in malaria low-transmission settings multigravidae continue to acquire protective immune responses to PM even after the second pregnancy. Hence, the objective of this study was to identify immune responses to VAR2CSA that were present in women who were PM- that were absent in multigravidae with PM. Plasma samples from 420 multigravid women who were PM+ (n = 96) and PM− (n = 324) were screened in 21 serological assays that measured IgG to full-length VAR2CSA (FV2); the six VAR2CSA DBL domains, including variants from different genetic background, proportion of Ab with high avidity to FV2 (i.e., per cent of Ab that remained bound to FV2 in the presence of 3 M NH_4_SCN), total number of DBL domains recognized, as well as Ab levels to non-pregnancy specific malarial antigens (MSP-1, MSP-2, AMA-1, RESA, CSP). Identifying fine immunological differences between women with PM and those without could expedite VAR2CSA-based vaccine development, a vaccine that could protect an estimated 85 million women and their fetuses worldwide from the severe effects of malaria [23].

## Methods

### Ethical consideration

The archival, coded samples used in the current study were exempt from human subject research by the Committee on Human Studies, University of Hawaii, Manoa (CHS#19912). The original studies were conducted according to the Helsinki Declaration principles and approved by the National Ethics Committee, Cameroon and the Institutional Review Board at Georgetown University. All participants gave written informed consent to use their blood samples to measure Ab to malaria.

### Study design and plasma samples

In this retrospective case–control study, archival plasma samples from a previous large cross-sectional study conducted between 1996 and 2001 [24, 25] were used. All the samples were collected at delivery from Cameroonian women living in Yaoundé. Yaoundé, the capital of Cameroon, is a malaria-endemic area, where entomological inoculation rates are estimated to be 13 infectious bites per person per year [26, 27]. Since the samples were collected before implementation of IPT and long-lasting insecticide treated bed nets, all of the women were likely to have become infected several times during pregnancy. Although the human immunodeficiency virus (HIV) status of the women is unknown, the prevalence of HIV among pregnant women attending antenatal-care clinics in 2001 in urban areas in Cameroon is estimated to be 4.0–13.6 % [28], making it unlikely that HIV had a major effect on the results. Since *P. falciparum* transmission is low in Yaounde, plasma samples were screened for Ab to FV2 (FCR3 strain) (see cut-off based on Cameroonian males in Additional file 1), to confirm that the women had become infected and produced Ab to FV2. Only plasma samples from women who were seropositive to FV2 FCR3 were further studied. The following inclusion criteria were used: multigravidae (≥3 pregnancies), 18 years or older, singleton live deliveries with babies that were >28 weeks of gestation, and had Ab to FV2. All multigravidae who fit the inclusion criteria and had PM were included in the study (n = 96). These multigravidae should have acquired immunity to PM during their previous ≥2 pregnancies, however, since they had PM it is likely they had not developed a complete protective immune response. For comparison, about three times the number (n = 324) of PM-negative multigravidae that met the inclusion criteria were randomly selected. Archival plasma samples from American pregnant women (n = 42) were used to establish the cut-off for seropositivity to non-pregnancy-specific malaria antigens. Twenty Cameroonian male plasma samples were used to establish cut-off for seropositivity to VAR2CSA antigens (cut-off for FV2 FCR3 was 2,326 median fluorescence intensity (MFI), cut-off for other antigens is presented in Additional file 1). Pre-term deliveries were defined as less than 37 weeks of gestation; low birth weight was defined as less than 2500 g.

### Diagnosis of placental malaria and anaemia

Thick and thin blood smears were prepared using maternal peripheral and placental intervillous space blood, and impression smears were made from biopsies of placental tissues. Slides were stained with Diff-Quick (Polysciences, Warrington, PA, USA, Cat no: 24606-250) and read by two microscopists to determine parasitaemia. Placental biopsies were also fixed in buffered formalin, embedded, stained with haemotoxylin-eosin, and examined for parasites. A woman was considered PM-positive (PM+) if IE were detected in blood smears of intervillous space blood, impression smears of villous tissue, or histological sections of the placenta [18]. Maternal peripheral blood was used to determine the haematocrit/packed cell volume (PCV). Using the definition of the World Health Organization, a woman was considered anaemic if the haematocrit/PCV was less than 30 % [25].

### Recombinant proteins

The panel of recombinant proteins of different regions of VAR2CSA have been described previously [18], and included: full-length VAR2CSA (FV2), DBL1 + 2, DBL 2, DBL 3, DBL 4, DBL 5, DBL 6 of the FCR3 strain expressed in Baculovirus-transfected insect cells at the University of Copenhagen, Denmark; DBL1, DBL3, DBL4, DBL5, and DBL6 for the 7G8, 3D7 and IT4 strains expressed in *Pichia pastoris* from J D Smith and colleagues (Seattle Biomedical Research Institute, USA) [29], and DBL 3 (A4 expressed in *Escherichia coli* provided by K Singh (NIAID, NIH, Bethesda, MD, USA). The FCR3 and IT4 parasite lines are isogenic due to historical contamination [30] and therefore designated in the manuscript as FCR3/IT4. In addition, non-pregnancy-associated malarial antigens were used, including recombinant AMA-1 3D7 (expressed in yeast), MSP-1_42_ 3D7 (*Escherichia coli*), and MSP-2 3D7 were provided by C Long, Malaria Vaccine Development Branch (MVDB), National Institute of Allergy and Infectious Disease, National Institutes of Health, Rockville, MD, USA. Synthetic peptides containing B cell epitopes from CSP and RESA were synthesized by AnaSpec, Inc. (San Jose, CA, USA) and reported previously in detail [31].

### Coupling of recombinant proteins to SeroMAP microspheres

The method used for coupling was reported previously [31]. Proteins were coupled at optimal concentrations to 1 million microspheres: DBL1-DBL6 at 1 µg, FV2 at 3 µg, AMA-1 3D7 and MSP-1 3D7 at 1 µg, RESA at 40 µg, CSP at 15 µg and MSP-2 3D7 at 0.2 µg.

### Measuring IgG using a multi-analyte platform assay

The multi-analyte platform (MAP) assay was performed as previously described [18, 19, 31]. Briefly, 50 µl of antigen-coupled microspheres (2000 microspheres/test) were incubated with 50 µl of a 1:100 dilution of plasma in phosphate buffered saline (PBS) containing 1 % bovine serum albumin (BSA) in pre-wetted filter plates (96-well Multiscreen BV; Millipore, Billerica, MA, USA), for 1 h at 25 °C on a rotating shaker at 500 rpm (Microplate Shaker, Lab-line, Melrose Park, IL, USA). Microspheres were washed twice with PBS-0.05 % Tween20 and once with PBS-1 % BSA. Then, 100 µl of secondary Ab (R-phycoerythrin-conjugated, Affini Pure F(ab′)_2_ fragment, Goat anti-human IgG Fc fragment specific, Jackson Immunoresearch, West Grove, PA, USA, Cat no. 109-116-170) diluted to 2 µg/ml in PBS-1 % BSA was added to each well and incubated as above in the dark for 1 h. Wells were washed as described above, microspheres were re-suspended in 100 µl PBS-1 % BSA and the microsphere suspension was analysed using a Liquichip M100 reader (Qiagen, Valencia, CA, USA). The reader was programmed to read a minimum 100 beads per spectral address, DD Gate 7500–15,000 and 35-s timeout. The results were expressed as MFI. Positive and negative controls were included on each plate consisting of three different pools of plasma from eight Cameroonian multigravidae with high Ab levels to VAR2CSA and pools of plasma from 40 Americans who had never travelled to malaria-endemic areas.

### IgG subclass determination to the FV2

For IgG subclass assay, each plasma sample was tested in quadruplicates. After initial incubation of FV2-coupled microspheres with plasma and washing as described above, microspheres were incubated with 100 µL mouse anti-human Ab to detect specific IgG subclasses: (a) mouse anti-human IgG1 (Molecular Probes, Ref. A10630) 1:1000 dilution, (b) mouse anti-human IgG2 (Sigma, ascites HP6014 I5635) 1:1000 dilution, (c) mouse anti-human IgG3 (Sigma, clone HP6050 I7260) 1:2000 dilution, and, (d) mouse anti-human IgG4 (Sigma HP6023 I9888) 1:1000 dilution. Plates were incubated for 1 h, at 25 °C on the shaker. After washing as described above, microspheres were resuspended in 100 µl of R-phycoerythrin-conjugated donkey anti-mouse IgG H + L (Jackson 715-116-150) at 1:250 dilution. Plates were incubated for 1 h, at 25 °C on a shaker, washed as before and resuspended in 100 µl PBS-1 % BSA; 85 µl of the microsphere suspension was analysed using a Liquichip M100 reader (Qiagen, Valencia, CA, USA). To test the quality of mouse-anti-human Ab, beads coupled to human monoclonal IgG1k, human monoclonal IgG2k, human monoclonal IgG3λ and human monoclonal IgG4λ were used. These beads were assayed as described above. Each sub-class was highly specific with no significant cross-reactivity with other IgG sub-classes (Additional file 2).

### Avidity to the FV2

The avidity assay was performed as previously described [19]. Briefly, plasma was diluted 1:300, 1:1000 and 1:3000 in PBS-1 % BSA and 50 µl of diluted plasma was added to six wells (each dilution in duplicate) containing 50 µl of FV2-coupled microspheres (2000 microspheres/test) and incubated for 1 h on a shaker at 25 °C. After incubation, 100 µl of 3 M NH_4_SCN in PBS-% BSA was added to half of the wells and 100 µl of PBS-1 % BSA was added to the other half (matching control wells for each dilution). After 30 min of incubation, the wells were washed as above and incubated with secondary Ab. Secondary Ab was washed as above and beads resuspended in 100 µl PBS-1 % BSA and analysed by MicroChip 100 as described in the MAP assay. Proportion Ab that remained bound (Ab avidity) in the presence of 3 M NH_4_SCN were determined for each dilution by the following formula: (MFI obtained from wells incubated with salt)/(MFI obtained from corresponding control wells) × 100 %, and the average was taken across three dilutions. Positive control consisting of pool of plasma from multigravida Cameroonian women and negative control consisting of pool of American plasma were included on each plate. Based on data from a previous study [19] when ≥35 % of Ab remained bound in the presence of 3 M NH_4_SCN, women were classified as having high avidity Ab.

### Determining the breadth of the VAR2CSA antibody response

The panel included 15 recombinant variants from three different strains (i.e., 3D7, FCR3, 7G8) that represent the six VAR2CSA DBL domains. A woman was considered to be Ab-positive for each of the 15 antigens if her plasma had a MFI greater than the mean MFI plus two standard deviations obtained with 20 plasma samples from Cameroonian males (unique cut-off was used for each antigen, see Additional file 1). In determining the breadth of the Ab response (repertoire), the total number of variants a woman recognized was determined, giving a score of 0–15 variants (note: DBL1 + 2 was excluded from this analysis, since it encompasses two DBL domains). A woman was considered Ab-positive for a particular domain, if she had Ab to one or more variant of that domain, i.e., giving a breadth of DBL domains of 0–6. Mean Ab levels plus two standard deviations of American controls was used as a cut-off for seropositivity to non-pregnancy-specific malarial antigens.

### Statistical analysis

Demographic, clinical and assay variables were summarized with means and standard deviations for the continuous variables, and frequencies and percentages for categorical variables. PM+ and PM− women were compared using both parametric and non-parametric approaches. As results were consistent, only parametric results (e.g., two sample t-tests with unequal variances for continuous variables and Chi square tests with a continuity adjustment, or Fisher’s exact, when applicable, for categorical variables) were reported. Finally, logistic regression models were developed to assess the association between PM status and each Ab response with and without adjustment for demographic and/or clinical variables. For ease of result interpretation, the assay data (MFI) were rescaled by interquartile range (IQR, the difference between 75th and 25th percentiles). All statistical analyses were performed using SAS 9.4 and GraphPad Prism; p value less than 0.05 was regarded as statistically significant.

## Results

### Characteristics of study population

The study population is described in Table 1. PM+ women were on average of 2 years younger (p = 0.0012); PM+ women on average had 4.5 ± 1.6 pregnancies, while PM-negative women had 5.2 ± 2.0 pregnancies (p = 0.0002). Seventy-one per cent (68/96) of PM+ women were also peripheral blood smear positive. Mean placental parasitaemia determined by impression smears was 3.5 % (7.6 %). Haematocrit levels were significantly lower in PM + women (<0.0001), resulting in a higher proportion of PM+ who were anaemic at delivery (p = 0.0055). No significant differences were observed between PM+ and PM− multigravidae in terms of prevalence of pre-term deliveries or neonate birth weight.

**Table 1:** Characteristics of pregnant women and their neonates

Characteristic^a^	PM positive (n = 96)	PM negative (n = 324)	p value
Age in years (mean ± SD)	27.6 ± 4.6	29.4 ± 5.6	*0.0012*
Gravidity (mean ± SD)	4.5 ± 1.6	5.2 ± 2.0	*0.0002*
Prevalence of women with peripheral malaria by blood smears, n (%)	68 (70.8 %)	5 (1.5 %)	*<0.0001*
Peripheral malaria parasitaemia among all women, parasites/µL (mean ± SD)	0.2 ± 0.6	0.0 ± 0.02	*0.0011*
Peripheral malaria parasitaemia among blood smear positive women parasites/µL (mean ± SD)	0.3 ± 0.7	0.1 ± 0.5	0.12
Prevalence of women with placental parasitaemia, n (%)	96 (100 %)	0 (0 %)	
Placental parasitaemia by impression smears, (%) (mean ± SD)	3.5 ± 7.6	0 ± 0	
Pre-term deliveries, n (%)	16 (16.7 %)	63 (19.4 %)	0.59
Length of pregnancy in weeks (mean ± SD)	39.1 ± 2.7	39.1 ± 3.0	0.95
Full-term deliveries	39.9 ± 1.7	40.3 ± 1.5	0.12
Pre-term deliveries	34.3 ± 1.7	34.0 ± 2.4	0.50
Intra-uterine growth restriction, n (%)	2 (2.1 %)	9 (2.8 %)	0.99
Maternal anaemia at delivery, n (%)	24 (25.0 %)	39 (12.0 %)	*0.0055*
Haematocrit in % (mean ± SD)	32.0 ± 5.0	34.7 ± 5.1	*<0.0001*
Low birth weight babies, n (%)	11 (11.5 %)	30 (9.3 %)	0.68
Among full-term deliveries	2 (2.5 %)	8 (3.2 %)	1.00
Among pre-term deliveries	8 (53.3 %)	20 (33.9 %)	0.30
Neonate weight in grams (mean ± SD)	3139 ± 600	3257 ± 611	0.10
Full-term deliveries	3303 ± 433	3393 ± 478	0.13
Pre-term deliveries	2337 ± 667	2681 ± 739	0.10

^a^For the univariate analyses, two-sample t tests for continuous variables and Chi square or Fisher’s exact tests for categorical variables were used to compare women in PM+ and PM-

*p* values that were less than 0.05 were designated in italics

### Differences in immune responses to VAR2CSA between multigravidae with and without PM

Multigravidae with PM and without PM had similar Ab levels to DBL1 + 2 (p = 0.37), DBL2 (p = 0.53), DBL3 (p = 0.32), DBL4 (p = 0.78), DBL5 (p = 0.54), DBL6 (p = 0.54), and FV2 (p = 0.76) from the FCR3 strain and on average recognized similar number of domains (4.2 ± 1.3) and variants (9.3 ± 3.7) (Table 2). Similar data were obtained for the other strain variants (Table 2). However, PM+ women had significantly lower avidity to FV2 (p = 0.0030) compared to multigravidae without PM, although the prevalence of women with high Ab avidity (≥35 %) was not statistically significant between the two groups (Table 2). Ab to non-pregnancy-specific malarial antigens were associated with infection: AMA-1 (p < 0.0001), MSP1 (p = 0.035), and MSP2 (p = 0.0028).

**Table 2:** Antibody response to malaria antigens in pregnant women at delivery

Antibody response^a^	PM positive (n = 96)	PM negative (n = 324)	p value
DBL1 3D7 (*Pichia pastoris*) (mean ± SD)	4832 ± 4487	4569 ± 4031	0.61
DBL1 7G8 (*Pichia pastoris*) (mean ± SD)	6479 ± 4095	6558 ± 4237	0.70
DBL1 + 2 FCR3 (Baculovirus) (mean ± SD)	8582 ± 6649	7901 ± 5996	0.37
DBL2 FCR3 (Baculovirus) (mean ± SD)	2416 ± 2709	2622 ± 3041	0.53
DBL3 FCR3 (Baculovirus) (mean ± SD)	6917 ± 5052	7524 ± 5675	0.32
DBL3 7G8 (*Pichia pastoris*) (mean ± SD)	9940 ± 6437	10,986 ± 6661	0.17
DBL3 FCR3 (*Escherichia coli*) (mean ± SD)	7535 ± 5542	8171 ± 5977	0.34
DBL4 FCR3 (Baculovirus) (mean ± SD)	7966 ± 4666	8130 ± 5481	0.78
DBL4 7G8 (*Pichia pastoris*) (mean ± SD)	4476 ± 4130	4594 ± 4510	0.73
DBL4 FCR3/IT4 (*Pichia pastoris*) (mean ± SD)	6274 ± 4333	5296 ± 4428	0.056
DBL5 FCR3 (Baculovirus) (mean ± SD)	15,749 ± 6804	15,270 ± 6213	0.54
DBL5 3D7 (*Pichia pastoris*) (mean ± SD)	13,237 ± 5857	12,124 ± 5727	0.11
DBL5 7G8 (*Pichia pastoris*) (mean ± SD)	13,043 ± 5563	12,403 ± 5524	0.33
DBL6 FCR3 (Baculovirus) (mean ± SD)	7,655 ± 5726	8078 ± 6287	0.54
DBL6 7G8 (*Pichia pastoris*) (mean ± SD)	7,293 ± 4953	7459 ± 4486	0.77
DBL6 FCR3/IT4 (*Pichia pastoris*) (mean ± SD)	4,364 ± 3473	4609 ± 3577	0.55
FV2 FCR3 (Baculovirus) (mean ± SD)	11,280 ± 5293	11,475 ± 5654	0.76
Per cent high avidity to FV2 (mean ± SD)	29.3 ± 12.4	33.7 ± 12.6	*0.0030*
Per cent of women with ≥35 % high avidity Ab (%)	37.5 %	46.9 %	0.11
Number of domains recognized out of 6 (mean ± SD)	4.2 ± 1.3	4.1 ± 1.3	0.65
Number of variants recognized out of 15 (mean ± SD)	9.3 ± 3.7	9.1 ± 3.6	0.67
CSP (mean ± SD)	920 ± 1030	974 ± 1066	0.66
RESA (mean ± SD)	1033 ± 2094	1047 ± 1861	0.96
AMA-1 3D7 (mean ± SD)	14,814 ± 4477	12,004 ± 5083	*<0.0001*
MSP1 3D7 (mean ± SD)	9051 ± 6976	7360 ± 6246	*0.035*
MSP2 3D7 (mean ± SD)	16,224 ± 8444	13,224 ± 8658	*0.0028*

^a^For the comparison between PM+ and PM− women in antibody response, two sample t tests for continuous variables and Chi square tests for categorical variables were used

*p* values that were less than 0.05 were designated in italics

To test whether VAR2CSA IgG sub-class distribution differed between women with and without PM, VAR2CSA IgG1, IgG2, IgG3, and IgG4 levels were measured in a sub-set of randomly selected 20 PM+ and 20 PM− women. Both PM+ and PM− multigravidae predominantly had only IgG1 to FV2 (Additional file 3).

Of note, seven out of twenty Cameroonian males were seropositive for VAR2CSA domains (data not shown). One male was seropositive for DBL1, DBL4, DBL5, DBL6 and FV2 FCR3, another male for DBL1 + 2, DBL3, DBL4, remaining five males were seropositive for one or two domains only. There was no single domain that was consistently cross-reactive in majority or all males, rather domain(s) to which Cameroonian males were seropositive to varied between individuals. Seven males recognized total of 19 domain variants, compared to women’s average for that particular domain: 13/19 had low Ab levels and additional individual had low Ab levels to FV2 FCR3, 2/19 had medium Ab levels and 4/19 had high Ab levels.

### Association between Ab to VAR2CSA and absence of PM

To test whether lack of particular Ab responses to VAR2CSA were associated with absence of PM, a logistic regression models was used (Table 3). The logistic regression models showed no increase with protection from absence of PM at delivery with high Ab levels to individual domains, thus confirming results in Table 2. However, higher avidity Ab to FV2 were significantly associated with the absence of PM (p = 0.0033); i.e. a 5 % increase in Ab avidity to FV2 was associated with a 15 % increase in the likelihood of a woman being protected from PM (95 % CI 1.01, 1.05) for the unadjusted model (Table 3). Ab avidity to FV2 remained significantly associated with protection from PM when logistic regression models were adjusted for gravidity only or gravidity and age (Table 3).

**Table 3:** Association between antibodies to VAR2CSA with absence of placental malaria in 420 women

	Increment unit	Unadjusted		Adjusted^a^		Adjusted^b^	
		OR (95 % CI)	p value	OR (95 % CI)	p value	OR (95 % CI)	p value
Immune responses to VAR2CSA							
DBL1 3D7 (*Pichia pastoris*)	5147.5	0.93 (0.70, 1.22)	0.59	0.90 (0.67, 1.20)	0.47	0.91 (0.68, 1.21)	0.51
DBL1 7G8 (*Pichia pastoris*)	5543.8	0.94 (0.70, 1.27)	0.70	0.95 (0.70, 1.28)	0.73	0.95 (0.70, 1.29)	0.75
DBL1 + 2 FCR3 (Baculovirus)	9077.3	0.85 (0.61, 1.19)	0.35	0.87 (0.62, 1.22)	0.43	0.88 (0.63, 1.23)	0.46
DBL2 FCR3 (Baculovirus)	2087.0	1.05 (0.89, 1.25)	0.55	1.07 (0.90, 1.27)	0.48	1.08 (0.90, 1.29)	0.40
DBL3 FCR3 (Baculovirus)	8125.0	1.18 (0.84, 1.67)	0.89	1.22 (0.86, 1.72)	0.27	1.23 (0.87, 1.75)	0.25
DBL3 7G8 (*Pichia pastoris*)	11,573.0	1.33 (0.88, 1.99)	0.18	1.28 (0.85, 1.93)	0.24	1.27 (0.85, 1.92)	0.25
DBL3 FCR3 (*Escherichia coli*)	9346.8	1.19 (0.82, 1.73)	0.36	1.16 (0.80, 1.68)	0.44	1.15 (0.79, 1.67)	0.46
DBL4 FCR3 (Baculovirus)	7023.0	1.04 (0.77, 1.41)	0.79	1.06 (0.78, 1.44)	0.73	1.07 (0.78, 1.46)	0.68
DBL4 7G8 (*Pichia pastoris*)	5244.3	0.96 (0.73, 1.25)	0.74	0.95 (0.72, 1.24)	0.69	0.94 (0.72, 1.24)	0.68
DBL4 FCR3/IT4 (*Pichia pastoris*)	5088.3	0.79 (0.61, 1.01)	0.059	0.80 (0.62, 1.03)	0.080	0.80 (0.63, 1.03)	0.086
DBL5 FCR3 (Baculovirus)	11,083.0	0.88 (0.59, 1.31)	0.52	0.88 (0.59, 1.32)	0.54	0.87 (0.58, 1.30)	0.49
DBL5 3D7 (*Pichia pastoris*)	9502.3	0.72 (0.49, 1.06)	0.098	0.72 (0.49, 1.06)	0.097	0.71 (0.48, 1.04)	0.079
DBL5 7G8 (*Pichia pastoris*)	9335.5	0.82 (0.55, 1.21)	0.32	0.81 (0.55, 1.21)	0.31	0.80 (0.54, 1.19)	0.27
DBL6 FCR3 (Baculovirus)	9534.0	1.11 (0.78, 1.60)	0.56	1.09 (0.76, 1.56)	0.66	1.09 (0.76, 1.56)	0.66
DBL6 7G8 (*Pichia pastoris*)	6554.5	1.05 (0.76, 1.46)	0.76	1.08 (0.78, 1.50)	0.66	1.05 (0.76, 1.47)	0.76
DBL6 FCR3/IT4 (*Pichia pastoris*)	3803.5	1.08 (0.84, 1.39)	0.56	1.05 (0.82, 1.36)	0.69	1.05 (0.82, 1.35)	0.72
FV2 FCR3 (Baculovirus)	9579.5	1.06 (0.72, 1.57)	0.77	1.06 (0.71, 1.57)	0.80	1.05 (0.71, 1.57)	0.81
Per cent high avidity to FV2	1	1.03 (1.01, 1.05)	*0.0033*	1.03 (1.01, 1.05)	*0.0079*	1.03 (1.01, 1.04)	*0.010*
Per cent of women with ≥35 % high avidity Ab to FV2	1	1.47 (0.92, 2.35)	0.11	1.45 (0.90, 2.32)	0.13	1.43 (0.89, 2.30)	0.15
Number of domains recognized out of 6	1	0.96 (0.81, 1.14)	0.65	0.96 (0.81, 1.15)	0.67	0.96 (0.81, 1.15)	0.68
Number of variants recognized out of 15	1	0.99 (0.93, 1.05)	0.67	0.98 (0.92, 1.05)	0.56	0.98 (0.92, 1.05)	0.58
Immune responses to non-pregnancy associated antigens							
CSP	875.5	1.05 (0.86, 0.27)	0.66	1.00 (0.82, 1.22)	1.00	1.01 (0.83, 1.24)	0.93
RESA	512.5	1.02 (0.94, 1.07)	0.95	1.00 (0.94, 1.06)	0.90	1.00 (0.94, 1.06)	0.90
AMA-1 3D7	7905.8	0.36 (0.23, 0.56)	*<0.0001*	0.37 (0.24, 0.58)	*<0.0001*	0.37 (0.24, 0.57)	*<0.0001*
MSP1 3D7	11,844.8	0.62 (0.41, 0.94)	*0.025*	0.60 (0.39, 0.92)	*0.019*	0.61 (0.40, 0.94)	*0.025*
MSP2 3D7	18,300.8	0.47 (0.29, 0.78)	*0.0032*	0.47 (0.28, 0.78)	*0.0034*	0.47 (0.28, 0.78)	*0.0038*

The antibody levels (MFI) were rescaled by IQR (the difference between 75th and 25th percentiles)

*p* values that were less than 0.05 were designated in italics

^a^Logistic regression models were adjusted for gravidity

^b^Logistic regression models were adjusted for gravidity and age

## Discussion

In this study, Cameroonian multigravidae in a low malaria transmission area acquired high Ab levels to both the N-terminal and C-terminal regions of VAR2CSA by the third pregnancy. The primary difference between multigravidae who were PM-negative was that they had fine-tuned their immune responses through affinity maturation as reflected by increases in the proportion of high avidity Ab to FV2 (p = 0.003) (Table 3). Examination of Ab levels to DBL domains and FV2, as well as breadth of response (number of domains and variants recognized) in multigravidae using a logistic regression model revealed that increase in Ab levels to DBL domains or FV2 was not associated with reduced likelihood of PM. On the other hand, an increase in the proportion of high avidity Ab to FV2 was significantly associated with reduced likelihood of PM at delivery in multigravidae (Table 3). This association continued to be significant even after adjusting model for gravidity and age.

Protection from PM is mediated by Ab to VAR2CSA. However, the exact mechanism of protection has not been elucidated. Currently available effector mechanisms of IgG to VAR2CSA include inhibition of binding [6, 8, 11, 32] and opsonic phagocytosis [5, 15, 17]. Another essential characteristic of Ab to VAR2CSA is Ab affinity. While affinity describes the interaction between a single Ab combining site and cognate antigenic determinant, avidity describes the overall strength of attachment of heterogeneous polyclonal IgG to multiple determinants on complex antigens. Data from previous study demonstrated that in high malaria transmission settings, the proportion of high avidity Ab to FV2 was significantly higher in PM− compared to PM+ women [19]. In the current study, results indicate that multigravidae living in low malaria transmission area, a 5 % difference in avidity of Ab to FV2 is associated with 15 % lower likelihood of PM in multigravidae (Table 3), while prevalence of women with PM is reduced with higher number of pregnancies.

Affinity maturation is an antigen-driven process [22, 33] and leads to enhanced B cells that progressively produce Ab with higher affinities and faster on-rates [22, 34]. A minority of B cells with affinity-enhancing mutations differentiate into long-lived Ab-secreting plasma cells and memory B cells. In diverse B cell receptor affinity population, B cells with high affinity B cell receptor have competitive advantage in binding to cognate antigen in an antigen-limiting environment. This view is in line with the findings that when immunogen is present in large doses the rate at which the affinity of serum Ab is increased is greatly reduced, since high levels of antigen on follicular dendritic cells reduce competitive advantage of high affinity B cell receptor [35].

Ab avidity data for VAR2CSA are not available for other countries. However, outside pregnancy, the notion that Ab quality is important for protection has been suggested [36], since similar IgG levels to *P. falciparum* were found in patients with severe and mild malaria [37]. High avidity Ab to an extract of *P. falciparum* schizonts (Amazonian parasite isolate) have been detected in clinically immune Senegalese adults [38]; high avidity Ab to blood stage antigen were observed among asymptomatic Brazilian adults [39]; affinity of Ab to AMA-1 and MSP2 increased with age and high affinity Ab to MSP2 were shown to prolong time to re-infection in Ugandan and Tanzanian children [40]. However, Ab avidity to AMA1, MSP1 and MSP3 were not associated with immunity to malaria among Kenyan [41] and Gambian children [42], possibly due to differences in techniques used and malaria transmission. In addition, Ab avidity can be important for mediating effector functions against placental-binding *P. falciparum,* such as inhibition of binding or opsonic phagocytosis. Humburger et al. demonstrated that protective nature of anti-malarial Ab could be reversed due to the low Ab avidity [43].

Of interest, Cameroonian pregnant women in this study had predominantly cytophilic IgG1 sub-class of Ab as reported previously [44]. IgG1 and IgG3 to variant surface antigen are associated with high opsonic phagocytosis activity of CSA-binding IE in Kenyan women; further, IgG1 and IgG3 levels to variant surface antigen on CSA-binding IE have strong positive correlation with phagocytic index [15]. In contrast, Guitard et al. found that VAR2CSA DBL5 in Senegalese pregnant women elicits high levels of both IgG1 and IgG3 [45].

In this study, several males living in Yaounde had Ab to VAR2CSA domains that were above cut-off (Additional file 1). This phenomena has been observed in previous studies conducted in rural Cameroonian villages with high malaria transmission (in press). It is likely that in individuals with high, chronic parasitemia, parasites switch to the VAR2CSA-expressing phenotype and induce an Ab response. For example, Ab levels to DBL5 have been reported in young Tanzanian children [46] with severe malaria and in more than 60 % Colombian men and children [47]. Another possibility is that conserved epitopes between VAR2CSA and other *Plasmodium falciparum* Erythrocyte Membrane P1 family antigens exist. However, results from a study by Beeson et al., who used plasma from men and children living in Papua New Guinea, Kenya and Malawi in a cell surface binding assay using both VAR2CSA-expressing and non-VAR2CSA expressing IE [48], showed that IgG-binding was specific and did not represent Ab to subpopulations of non-CSA-binding IE. Further, sera from some non-pregnant individuals inhibited IE adhesion to CSA. Hence, it is likely that IgG observed in non-pregnant individuals are specific to VAR2CSA and are not due to the cross-reactivity with non-VAR2CSA surface antigens.

This study had several limitations. One key limitation was a cross-sectional nature of the original study and therefore absence of data on clinical and malaria exposure of the pregnant women throughout pregnancy. Thus, stratification of women into better-defined groups based on their clinical histories was not possible. Additional limitations include: absence of PM categorization according to Bulmer score and only one form of FV2 was available for testing. Finally, information on functional properties of Ab to VAR2CSA were not included in this analysis. Currently, studies on opsonic phagocytosis and inhibition of binding to CSA are being carried out.

Finally, protection from PM depends on acquisition of Ab to VAR2CSA. It is not clear whether protection could be mediated by high titres and affinity maturation to a single conserved VAR2CSA epitope, perhaps within the CSA-binding site, during repeat infections or acquisition of a broad range of IgG to many variants (genotypes) of VAR2CSA, that is, protection would require Ab to ‘enough’ variants. Since in multigravidae total anti-VAR2CSA IgG levels did not increase with increasing gravidities, it is possible that CSA-binding parasites express ‘decoy’ conserved immunodominant epitopes, which elicit high IgG titres that are not associated with absence of PM. Multigravidae in the present study continued to have PM even though they had high IgG titers to various VAR2CSA domains, e.g., DBL3, DBL5 and DBL6. In addition, no single VAR2CSA domain tested was associated with absence of PM in this study. If protection from PM is mediated by a single conserved sequence or domain of VAR2CSA, Ab boosting to that sequence might be difficult to detect using the method employed in this study. Since individual recombinant DBL domains used in the study were “removed from the molecule,” it is also possible the conformational epitopes created by different domains in the intact antigen are critical for inducing protection. Overall, data reported in this study support the conclusion that Ab avidity to FV2 plays a role in immunity to PM.

Ab avidity is an important determinant of successful vaccination against infections such as H1N1 [49], Hib *Haemophiluys influenzae* type B [50] and *Bordetella pertussis* [51]. In addition, recent studies in African infants who received RTS,S/AS01E in phase 2 trial demonstrated that “change in avidity following second and third RTS,S/AS01E injection was associated with a 54 % likelihood reduction of getting malaria” [52]. Whether high avidity Ab to VAR2CSA can be induced by vaccination remains unknown.

## Conclusion

Results from this study suggest that high avidity Ab to VAR2CSA may play a key role in immunity to PM. It is conceivable that Ab avidity to FV2 is an important surrogate marker for assessing VAR2CSA vaccine efficacy.

## Additional files

10.1186/s12936-015-1023-6 Cut-off values for DBL domains and FV2.
10.1186/s12936-015-1023-6 Testing mouse anti-human monoclonal antibodies for cross-reactivity (median fluorescent intensities tabulated).
10.1186/s12936-015-1023-6 IgG sub-class levels to the full-length VAR2CSA. IgG sub-class levels to FV2 FCR3 were measured in randomly selected 20 PM+ and 20 PM-negative women. Median and interquartile ranges plotted.

## Abbreviations

Ab: antibodies
AID: activation-induced cytidine deaminase
BSA: bovine serum albumin
CSA: chondroitin sulfate A
DBL: VAR2CSA Duffy binding-like domains
FV2: full-length VAR2CSA
HIV: human immunodeficiency virus
IE: infected erythrocytes
IgG: immunoglobulin G
IPT: intermittent preventive treatment
MAP: multi-analyte platform assay
MFI: median fluorescent intensity
PBS: phosphate buffered saline
PCV: packed cell volume
PM: placental malaria
PM+: placental malaria positive
PM: placental malaria negative
