# Supplementary figures and images for: Comparison of the specificity of antibodies to VAR2CSA in Cameroonian multigravidae with and without placental malaria: a retrospective case–control study
Source: Malar J. 2015 Dec 1;14:480. doi: 10.1186/s12936-015-1023-6 (PMC4666123; doi:10.1186/s12936-015-1023-6)

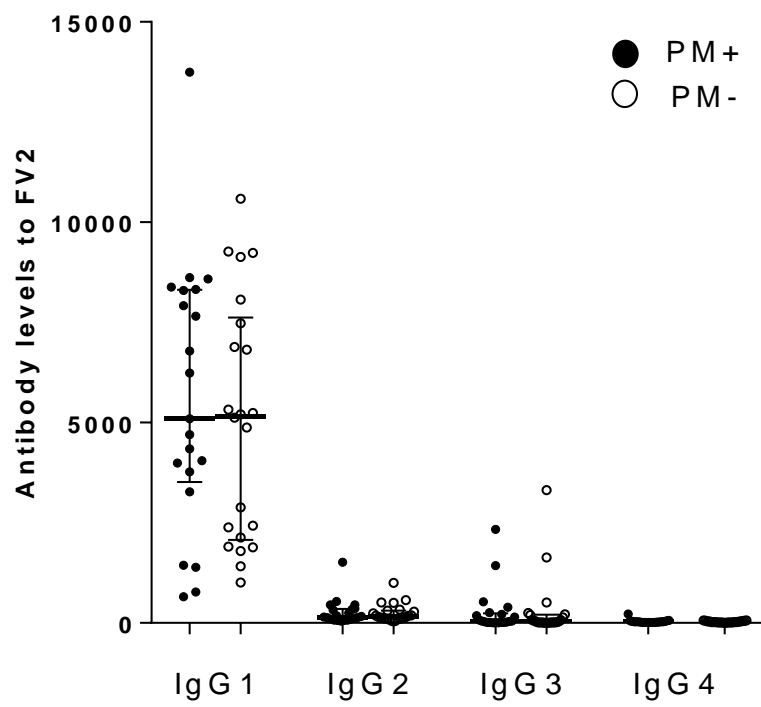

Supplement: Supplementary file 3 — 10.1186/s12936-015-1023-6 IgG sub-class levels to the full-length VAR2CSA. IgG sub-class levels to FV2 FCR3 were measured in randomly selected 20 PM+ and 20 PM-negative women. Median and interquartile ranges plotted. [file 12936_2015_1023_MOESM3_ESM.pdf]
